# Myosin Assembly, Maintenance and Degradation in Muscle: Role of the Chaperone UNC-45 in Myosin Thick Filament Dynamics

**DOI:** 10.3390/ijms9091863

**Published:** 2008-09-19

**Authors:** Torah M. Kachur, David B. Pilgrim

**Affiliations:** Department Biological Sciences, CW-405 Biological Sciences Building, University of Alberta, Edmonton, Alberta, Canada T6G 2E9

**Keywords:** Myosin, chaperone, protein folding, UNC-45, motor domain, heat shock protein

## Abstract

Myofibrillogenesis in striated muscle cells requires a precise ordered pathway to assemble different proteins into a linear array of sarcomeres. The sarcomere relies on interdigitated thick and thin filaments to ensure muscle contraction, as well as properly folded and catalytically active myosin head. Achieving this organization requires a series of protein folding and assembly steps. The folding of the myosin head domain requires chaperone activity to attain its functional conformation. Folded or unfolded myosin can spontaneously assemble into short myosin filaments, but further assembly requires the short and incomplete myosin filaments to assemble into the developing thick filament. These longer filaments are then incorporated into the developing sarcomere of the muscle. Both myosin folding and assembly require factors to coordinate the formation of the thick filament in the sarcomere and these factors include chaperone molecules. Myosin folding and sarcomeric assembly requires association of classical chaperones as well as folding cofactors such as UNC-45. Recent research has suggested that UNC-45 is required beyond initial myosin head folding and may be directly or indirectly involved in different stages of myosin thick filament assembly, maintenance and degradation.

## 1. Building the Sarcomere

**Figure 1.**
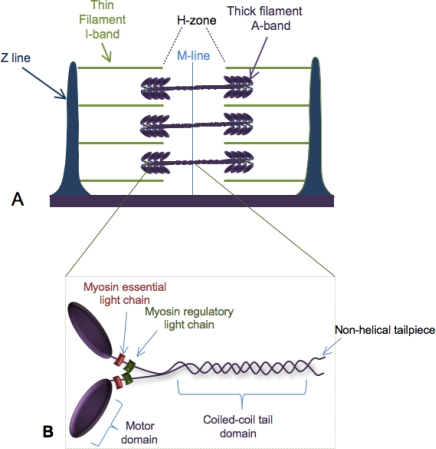
Myosin thick filaments in the sarcomere. A) Schematic of the sarcomere showing the relationship between the myosin-containing thick filaments and the actin-containing thin filaments. B) Type II conventional muscle myosin structure.

As will be familiar to most readers, the sarcomere is a near-crystalline array of proteins in muscle cells that exhibit a strict arrangement into Z-lines, I-bands and A-bands (Figure 1A). The main structural feature of the sarcomere is the interdigitated myosin-containing thick filaments (A-bands) and actin thin filaments (I-bands). The thick filament is a multiprotein complex composed mainly of myosin that assembles into antiparallel arrays and extends the full length of the thick filament providing the contractile force necessary during muscle contraction.

Myosin has many cellular roles besides that in muscle, and there are 18 different classes of myosins found amongst all organisms (reviewed in [1]), but the myosins required in the sarcomere are Type II myosins. Conventional type II myosins are hexamers characterized by a two-headed structure, a flexible neck region containing the myosin light chains and a long coiled-coil tail domain (Figure 1B). The functional myosin unit includes a myosin heavy chain dimer and two light chains per heavy chain; one regulatory and one essential light chain. In order for proper function of the thick filament, all regions of the myosin motor must be correctly folded and assembled.

Myosin can spontaneously assemble into thick filament-like structures *in vitro* but the myosin head domain cannot fold without muscle-specific factors, suggesting that this process requires the assistance of both general and myosin specific chaperones [2]. The myosin must also assemble along with other proteins into an organized bipolar filament with the tail domains associated into an antiparallel array with the head domains protruding out of the filament in a regular fashion. Although there are other critical components of the thick filament, this review will focus on the steps of myosin assembly from myosin head folding to maintenance and degradation in the sarcomere with a particular focus on the chaperones thought to be required to maintain myosin in a contractile form.

## 2. Myosin Folding

All classes of myosin share a catalytic domain of ∼750 amino acids that contains both the ATPase and actin-binding activities. The three dimensional structure of myosin is critical to its function and the proper folding of the head domain, in particular, is essential for the contraction of the sarcomere. The crystal structure of the Type II myosin head has been known for some time, where the core folding motif consists of seven mostly parallel, beta-pleated sheets that are flanked by three alpha-helices on each side to form the ATP binding pocket [3–5]. Despite the knowledge of the crystal structure of the myosin head, little is known about the folding kinetics of the myosin head domain except that head folding requires the assistance of specialized folding proteins called chaperones.

The folding of a protein is often a very complex process that is essential for proper function. The specificity of protein activity is dictated by its conformation and its folding pattern is promoted by the hydrophobic non-polar amino acids that avoid interactions with the aqueous cytosol. Chaperones can promote the proper folding of proteins by recognizing stretches of hydrophobic amino acids that are exposed upon translation or misfolding and can initiate one of three pathways: promoting proper folding by an ATP dependent mechanism, preventing aggregation by shielding the hydrophobic domains that would normally aggregate with hydrophobic domains of other proteins or targeting misfolded proteins for degradation by recruiting ubiquitinating enzymes and subsequent degradation by the 26S proteasome.

**Figure 2.**
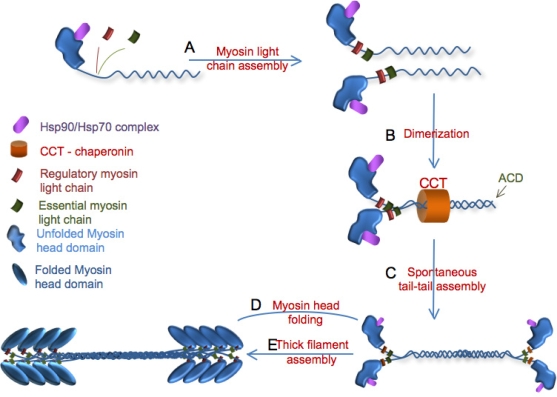
Myosin maturation. A) Nascent myosin molecules associate with the Hsp70/Hsp90 complex (purple). Myosin light chain assembly (green and red) and folding of the tail region occurs rapidly. B) Dimerization of the myosin tails may require the chaperonin CCT (orange). C) The assembly-competence domain (ACD) at the extreme C-terminus promotes tail-tail dimer formation. D) Myosin head folding is the rate limiting step and can occur at any point in this cascade. E) Assembly of myosin into thick filaments likely requires many different factors but does not require properly folded myosin heads. Note: the exact position of Hsp90 binding within the head domain is not known.

Many proteins require chaperones to attain their functional conformation; for example catalytically active myosin cannot be easily expressed in most *in vitro* systems because muscle specific chaperones are needed to fold the globular myosin head [6–9]. Chaperones such as heat shock protein 90 and 70 (Hsp90 and Hsp70 respectively) complex with all newly synthesized myosin molecules that contain a partially folded motor domain and are required for myosin head function (Figure 2A)[9]. However, Hsp70 and Hsp90 are ubiquitous chaperones present in all tissues, therefore specificity factors must be involved in folding the muscle myosin head *in vivo* in muscle cells.

### 2.1. Role of unc-45 in myosin folding

One important specificity factor for *hsp90*-mediated myosin folding is UNC-45 *(uncoordinated-45)* that was originally identified as a result of mutations causing structural disruption of thick filaments in body wall muscle in the nematode *Caenorhabditis elegans* [10, 11]. *unc-45* is an essential gene in *C. elegans,* but conditional alleles (missense mutations) result in disorganized and reduced numbers of myosin-containing thick filaments giving rise to a slow-moving (uncoordinated) phenotype of adult worms [12, 13]. UNC-45 homologs are present throughout metazoans and two homologs are found in the vertebrate genomes examined (humans, mice and teleost fish) [10, 14, 15]. In vertebrate model systems the two homologs of UNC-45 have different expression patterns and functions. *Unc45b* in zebrafish and mice is muscle-specific and is required for both cardiac and skeletal muscle function; when *Unc45b* is absent the myosin containing thick filaments in trunk muscles are reduced and disorganized [14, 16, 17]. The second vertebrate homolog, *Unc45a*, is ubiquitously expressed suggesting a more general role and has been implicated in a diverse array of activities from chaperoning the progesterone receptor [18, 19, 20] to cell proliferation and oncogenesis [14, 21], and most recently to defects in formation of the circulatory system [22].

UNC-45 acts as a chaperone for the myosin head domain in two biochemically distinct ways; 1) by serving as an activator for its co-chaperone, Hsp90 [23], and 2) by preventing the aggregation of misfolded myosin molecules [24]. Hsp90 acts within a large multisubunit chaperone complex that also contains Hsp70 and other co-chaperones but arrives later to the myosin folding complex to facilitate completion of head folding (23, 25 reviewed in [26]). Hsp90 alone is unable to catalyze any substantial folding changes in target proteins and requires association with co-chaperones that contribute to target specificity and folding (reviewed in [27]). Studies of the general cell isoform of UNC-45 (GCUNC45) and Hsp90 chaperone activity on the progesterone receptor have revealed that the GCUNC45 cochaperone binds the N-terminal domain of Hsp90 and prevents the ATPase activity of Hsp90 [19]. The binding of GCUNC45 may stall chaperone activity to allow for additional events to occur during the folding cascade of the progesterone receptor. It is unclear whether a similar biochemical function for UNC-45 occurs during myosin head folding. The Hsp90/UNC-45 co-chaperone complex has been found to be required for myosin function in zebrafish [17, 28, 29], mouse [23, 25] and nematode models [21] and the Hsp90-co-chaperone complex is likely required to induce subtle changes in the folding or arrangement of domains in substantially folded myosin [2].

Investigations of the biochemical function of UNC-45 in mouse cells have shown that *Unc45a* acts as an activator of the Hsp90 dependent protein folding activity of a type II smooth muscle myosin [23]. The function of UNC-45 in this complex may be to facilitate the interaction of the Hsp90 protein with the myosin head domain, providing a specificity and activation factor for Hsp90 during myosin head folding [2, 23, 24]. The interaction of Hsp90 with the myosin head allows Hsp90-dependent ATP hydrolysis to promote the conformational change in the substrate and eventual release of the correctly folded myosin [23]. Surprisingly, the striated muscle-specific homolog, *Unc45b,* can similarly promote smooth muscle myosin folding but at a lesser rate and efficiency than the general cell isoform. Although *Unc45b* is the muscle specific homolog, it was unable to facilitate folding with Hsp90 on a type II muscle myosin head suggesting that myosin head folding for muscle myosins requires additional factors [23, 25]. Therefore, UNC-45 is an essential part of the myosin head folding cascade by acting both as a specificity factor as well as an activator of Hsp90-mediated chaperone activity.

## 3. Myosin Assembly

There are at least three steps of myosin assembly that must occur prior to myosin function, including tail dimerization, antiparallel tail-tail association and thick filament assembly (Figure 2). All of these steps can occur without a properly folded myosin head domain (Figure 2A and B); therefore, head folding is the rate-limiting step of myosin formation and can occur at any one of the myosin assembly steps [2, 9]. Although myosin assembly can occur spontaneously *in vitro*, *in vivo* factors may accelerate assembly including a chaperonin and possibly chaperones such as UNC-45.

The first step of myosin assembly is dimerization of the myosin coiled-coil tails (Figure 2B) [9, 24]. The myosin rod domain contains a short-range 7 amino acid repeat containing four hydrophobic amino acids and a long-range 28 amino acid repeat that is characteristic of coiled-coil proteins (reviewed in [6]). Dimerization occurs spontaneously because of the maximization of charge interactions between amino acids in this 28 amino acid repeat. The initiation of dimerization may require the chaperonin CCT [7]. Chaperonins are a class of chaperones that consist of a multisubunit ring structure that likely provide a sequestered environment that favors folding [30].

The second assembly step is the formation of tail-tail antiparallel myosin dimers that can nucleate thick filament formation; this step is also spontaneous and requires the assembly competence domain (Figure 2C) [6]. The assembly competence domain (ACD) is a conserved 29 residue sequence at the C-terminus of the sarcomeric myosin rod domain. This includes a conserved set of 4 negatively charged amino acids is surrounded by positively charged amino acids [31]. The ACD allows the tails of myosin dimers to self-assemble into antiparallel arrays that form the centre H-band of the thick filament. Filaments are rarely composed of only one form of type II myosin and often differences in the distribution of myosins within the thick filament are a result of subtle yet unidentified sequence differences in the tail domain [32, 33]. For example, nematode body wall muscle thick filaments are composed of two different myosin heavy chains (MHC), A and B [34]. The myosin heavy chains are localized to different regions of the thick filament in *C. elegans*; MHC A is restricted to the central 2μm region of the sarcomere whereas MHC B is present along the majority of the sarcomere but is excluded from the central region [35]. The biochemical explanation for this localization pattern is that MHC A, but not B, contains a non-helical tailpiece that may promote antiparallel dimerization in order to localize to the antiparallel central region of the thick filament. However, the non-helical tailpiece is not required for the early events of myosin assembly [36]. The composition of thick filaments and the assembly of these different components into distinct domains within the thick filament remains poorly understood.

Myosin assembly into the thick filament complex requires the formation of large myosin filaments that contain thousands of individual myosin molecules (Figure 2E). The ability to assemble into thick filament-like structures is an intrinsic property of myosin and can occur spontaneously *in vitro* but likely involves association of other factors like titin *in vivo* that may help regulate thick filament structure and length [37]. Once bipolar myosin dimers are formed these complexes begin association into nascent thick filaments along with other components such as nebulin and some species-specific factors like paramyosin in *C. elegans* [6]. The mechanism of assembly of the intact thick filament into the sarcomere is unclear; currently four different sarcomeric assembly hypotheses have been proposed [38] that differ in the state of the thick filament prior to sarcomere formation.

### 3.1. Role of UNC-45 in myofibril assembly

**Figure 3.**
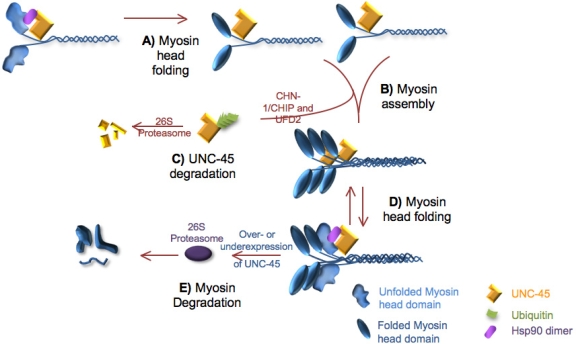
Roles for UNC-45. A) UNC-45 and the Hsp90 co-chaperone are responsible for folding the globular myosin head domain before or after myosin assembly. B) UNC-45 may promote myosin assembly into thick filaments. C) UNC-45 levels are tightly regulated by ubiquitination to ensure accurate myosin assembly. D) UNC-45 and Hsp90 are required to shuttle between the Z-disks and A bands possibly to refold the head domains that have unfolded during contraction. E) Under-and overexpression of UNC-45 in the adult muscle results in myosin degradation by the UPS. Note: For simplicity, myosin light chains have been omitted in this diagram.

In the nematode model, UNC-45 was shown to localize specifically with MHC B and not MHC A in thick filaments, implying a specific role with MHC B as opposed to MHC A [13]. Additionally, UNC-45 protein fails to localize properly in muscle lacking MHC B; for instance, in MHC B null mutant backgrounds [13]. These results suggest that UNC-45 may play a role as an assembler for thick filament formation (Figure 3B) specifically with loading of MHC B onto the nascent thick filament nucleated by MHC A tail-tail dimerization.

The hypothesis of an UNC-45 requirement in thick filament assembly was first provided by isolation of a temperature sensitive mutant. *unc-45(ts)* mutations had the most severe effect when gene function is reduced at the larval stage where myosin assembly is taking place, as opposed to in adulthood [10]. UNC-45 is most highly expressed at the L4/adult transition and once the worm reaches adulthood the somatic levels of UNC-45 drop off considerably [39]. The disappearance of UNC-45 in the adult muscle is due to rapid degradation by the ubiquitin proteasome system where UNC-45 is ubiquitinated by two ubiquitin conjugation factors, CHN-1 and UFD-2 (Figure 3C) [40]. The paralysis that is observed in some systems resulting from overexpression of UNC-45 may be a consequence of interference with the progression of myosin head folding. No obvious role has been found for wildtype levels of UNC-45 in the adult muscle; this, coupled with the decrease in UNC-45 levels in the adult suggests that UNC-45 is required primarily during initial assembly and growth of thick filaments and not maintenance in the adult worm muscle (reviewed in [41]).

## 4. Maintenance of the Thick Filament

Muscle cells are long-lived in both vertebrates and invertebrates and maintenance of the sarcomeric proteins is essential for continued muscular contraction. However the physical dynamics of contraction may result in a variety of conditions that produce stresses within the cell, including increased temperature [42], membrane lesions [17] and cell tension [43]. Cell stresses such as tensile forces have been shown to cause unfolding of the myosin head domain in non-muscle cells, raising the possibility that muscle myosins could also be sensitive to force-induced unfolding during muscular contraction [43]. Chaperones can be used to resolve any stress-induced misfolding that occurs in both muscle and non-muscle cells by remaining localized with or near the target client protein (Figure 3D). Chaperone function and localization within the assembled sarcomere has been observed. For example, alphaB crystallin is a member of the small heat shock protein chaperone family that associates with titin at the I-band of muscles during ischemia [44]. alphaB crystallin can move from the cytosol to the myofibrils following cell stress [45, 46] and mutations affecting the function of alphaB crystallin can cause defects in cardiac muscle function such as dilated cardiomyopathy [47]. Therefore, chaperone activity within the functioning sarcomere is essential for maintenance of contraction.

The chaperone UNC-45 remains associated with fully functional, and folded, myosin in the body wall muscle of *C. elegans* [13] suggesting that UNC-45 may still be required in the assembled sarcomere, possibly to resolve stress-induced misfolding. It was recently demonstrated that the skeletal muscle ortholog in zebrafish (*Unc45b*) shuttles from the Z-disks to the myosin thick filaments (A-band) of the sarcomere when the muscle cell has been stressed [48]. These early results could suggest that the dynamic movement of *Unc45b* during cell stress may represent a role for *Unc45b* in thick filament maintenance in zebrafish muscles.

In *C. elegans*, UNC-45 strongly co-localizes with MHC B (but not with another muscle myosin heavy chain, MHC A) and a dynamic movement within the sarcomere has not been observed. Similarly, UNC-45 remains associated with a type II non-muscle myosin, NMY-2, at the cortex of nematode embryos that is in stark contrast to the fold-and-release function of the majority of folding chaperones [49]. However, at least in muscle cells, the C-terminal UCS domain interacts with the neck region of type II muscle myosins (D.P. unpublished) but the neck region is unlikely to require chaperone activity in folding [7]. This localization pattern may reflect a different strategy in worms to address the same need of keeping UNC-45 close in case of myosin head unfolding.

## 5. Turnover of the Thick Filament Components

Turnover of sarcomeric components is essential in muscle cells whose lifespan far exceeds the half-life of the individual protein components; this obviously necessitates degradation of the myosin heavy chains. One of the major mechanisms to degrade cellular proteins is the ubiquitin proteasomal system (UPS) where proteins are targeted for degradation by the addition of polyubiquitin chains by a series of E1, E2, E3 and possibly E4 ubiquitin ligases. Once ubiquitinated the proteins are then degraded by 26S proteasome-mediated proteolysis. The proteasomal degradation mechanism is very active in muscle cells, to degrade muscle cell proteins including components of the sarcomere, and particularly in disease states [41, 50–52]. Myosin heavy chain is degraded by the UPS but when myosin is present as large myofibrillar complexes this degradation occurs very slowly [53]. Therefore, the UPS is mostly responsible for degrading myosin once it has been released from the thick filament and it is possible that cytosolic proteases cleave smaller myofilaments off the sarcomere to be rapidly degraded [reviewed in 50]. The factors that control myosin degradation in the muscle are keys to understanding muscular atrophy and a range of myopathies.

### 5.1. UNC-45 and myosin turnover

Myosins, and other unassembled sarcomeric components, are degraded by the UPS [52]. Both over-and under-expression of UNC-45 in *C. elegans* results in a decrease in the amount of assembled myosin [12, 54]. The model proposed in Landsverk *et al.* [54] suggests that precise levels of UNC-45 are required for promoting myosin folding and assembly and any deviation from these levels may prevent myosin from properly assembling thus triggering degradation of myosin by the UPS. However, no causal relationship between UNC-45 and ubiquitination of myosin was demonstrated, suggesting that UNC-45 may not be directly responsible for targeting myosin for ubiquitination. Additionally, no role for wild-type levels of UNC-45 has been found during myosin degradation. Although no direct link between UNC-45 and the degradation of myosin has been shown, the current data provides insight into a mechanism of myosin quality control that may be facilitated by its chaperone, UNC-45.

## 6. Human diseases of thick filament organization

Diseases such as myotonic dystrophy and other myopathies have been shown to require chaperones, possibly to facilitate assembly or to prevent aggregation of sarcomeric components [55]. There are many myopathies that result from mutations in sarcomeric proteins that include mutations in both of the major components of the sarcomere – actins and myosins (reviewed in [56]). Some of these myopathies have been suggested to be due to a loss of muscle organization or accumulation of aggregates. This includes a hyaline inclusion body myopathy that is characterized by aggregates of myosin likely caused by failed assembly of the thick filaments. Patients with these myopathic characteristics contain a missense mutation in a type II skeletal myosin (*MYH7*) in the tail domain of myosin that facilitates thick filament formation [57–59].

UNC-45 and CDC-48 have been shown to interact in human cell culture lines and CDC-48 has been implicated in inclusion-body myopathy associated with Paget disease of bone and frontotemporal dementia (IBMPFD) [39] suggesting that chaperones like UNC-45 may also play a significant role in muscle related illnesses. Additionally, as humans age, there is a marked decrease in muscle strength and efficiency. This gradual muscle weakness may be a result of decreased chaperone activity [60]. Part of this increase in muscle weakness may also be due to increased protein degradation of sarcomeric components during atrophy [61].

Arteriovenous malformations (AVM) are genetic abnormalities in the circulatory system that lead to hemorrhage, systemic hypoxia and stroke. In zebrafish, mutants in *Unc45a* display AVM by failing to properly form specific aortic arches, resulting in global circulation defects [22]. There are several genes known to cause AVMs in both mouse and human subjects but identification of a zebrafish model for human AVM formation allows for precise developmental timing and *in vivo* analysis of the causes of AVM. It is unknown whether a specific myosin is affected in these mutant fish or if this newly discovered phenotype represents another client protein for the UNC-45 chaperone complex.

The discovery of the role of chaperones such as *unc-45* during myosin assembly, turnover and degradation in both natural and pathological conditions suggests that chaperones provide a potential therapeutic target for muscle degeneration. By regulating chaperone activity, either by promoting efficient folding or by preventing misfolded aggregates, the medical community may be able to control the severity of myosin degeneration in the muscles of the body.

## 7. Conclusions

It is becoming increasingly clear that establishing the organization of the sarcomere and assembling the thick filament are complicated processes that are susceptible to errors. These errors could translate into myopathies and other diseases of muscle cell components. The chaperone UNC-45 is involved in the many different steps of thick filament formation, from the initial folding of the myosin head to the eventual degradation of myosin. The sole UNC-45 homolog in *C. elegans* and the two specialized orthologs in vertebrates represent a class of chaperones that function beyond simple nascent peptide folding but instead may function at all stages of myosin function from folding, thick filament maintenance and turnover.
